# Method for accurate multi-growth-stage estimation of fractional vegetation cover using unmanned aerial vehicle remote sensing

**DOI:** 10.1186/s13007-021-00752-3

**Published:** 2021-05-17

**Authors:** Jibo Yue, Wei Guo, Guijun Yang, Chengquan Zhou, Haikuan Feng, Hongbo Qiao

**Affiliations:** 1 College of Information and Management Science, Henan Agricultural University, Zhengzhou, 450002 China; 2 International Institute for Earth System Science, Nanjing University, Nanjing, 210023 China; 3 Key Laboratory of Quantitative Remote Sensing in Agriculture of Ministry of Agriculture, Beijing Research Center for Information Technology in Agriculture, Beijing, 100097 China; 4 Institute of Agricultural Equipment, Zhejiang Academy of Agricultural Sciences (ZAAS), Hangzhou, 310000 China

**Keywords:** Unmanned aerial vehicle, Fractional vegetation cover, Chlorophyll, Pixel dichotomy model, Soybean

## Abstract

**Background:**

Fractional vegetation cover (FVC) is an important parameter for evaluating crop-growth status. Optical remote-sensing techniques combined with the pixel dichotomy model (PDM) are widely used to estimate cropland FVC with medium to high spatial resolution on the ground. However, PDM-based FVC estimation is limited by effects stemming from the variation of crop canopy chlorophyll content (CCC). To overcome this difficulty, we propose herein a “fan-shaped method” (FSM) that uses a CCC spectral index (SI) and a vegetation SI to create a two-dimensional scatter map in which the three vertices represent high-CCC vegetation, low-CCC vegetation, and bare soil. The FVC at each pixel is determined based on the spatial location of the pixel in the two-dimensional scatter map, which mitigates the effects of CCC on the PDM. To evaluate the accuracy of FSM estimates of the FVC, we analyze the spectra obtained from (a) the PROSAIL model and (b) a spectrometer mounted on an unmanned aerial vehicle platform. Specifically, we use both the proposed FSM and traditional remote-sensing FVC-estimation methods (both linear and nonlinear regression and PDM) to estimate soybean FVC.

**Results:**

Field soybean CCC measurements indicate that (a) the soybean CCC increases continuously from the flowering growth stage to the later-podding growth stage, and then decreases with increasing crop growth stages, (b) the coefficient of variation of soybean CCC is very large in later growth stages (31.58–35.77%) and over all growth stages (26.14%). FVC samples with low CCC are underestimated by the PDM. Linear and nonlinear regression underestimates (overestimates) FVC samples with low (high) CCC. The proposed FSM depends less on CCC and is thus a robust method that can be used for multi-stage FVC estimation of crops with strongly varying CCC.

**Conclusions:**

Estimates and maps of FVC based on the later growth stages and on multiple growth stages should consider the variation of crop CCC. FSM can mitigates the effect of CCC by conducting a PDM at each CCC level. The FSM is a robust method that can be used to estimate FVC based on multiple growth stages where crop CCC varies greatly.

## Background

Fractional vegetation cover (FVC, sometime referred to as “crop canopy coverage”) is the fraction of green vegetation seen from the nadir of a study area and describes the fraction of the mixed vegetation versus soil in an ecosystem [1]. FVC is an important parameter for evaluating crop-growth status and is essential for crop-growth models [2–4]. Moreover, long-term FVC estimates are also essential for regional and global environmental monitoring because it is an essential indicator of dynamic changes in vegetation [5–9]. Thus, real-time estimates of FVC are of significant importance for both the agricultural and environmental research community.

Traditionally, photographic techniques have been widely used for measuring farmland FVC. Photographic techniques involve the use of classification techniques (e.g., the threshold method or classification tools) or artificial counting to analyze the FVC based on images of the field canopy [10–13]. However, such techniques are time and labor intensive and are difficult to exploit for FVC mapping.

Optical remote-sensing techniques collect surface radiation to provide crop-canopy spectral reflectance from visible to short-wave infrared wavelengths [14, 15]. In practice, leaf-pigment content and the leaf-area index (LAI) are the two main variables that determine the crop-canopy spectral reflectance [16–20]. Canopy chlorophyll content (CCC) and LAI govern the spectral reflectance in the visible bands, whereas LAI alone governs the spectral reflectance in the near-infrared (NIR) and short-wave infrared bands [16–19]. Leaf-chlorophyll absorption causes crop spectral reflectance in the blue and red bands to be less than that in the NIR band [21].

Many remote-sensing spectral indices (SIs) have been developed to quantify vegetation states [22]. A remote-sensing SI combines the vegetation canopy spectral reflectance in two or more bands, and one of the most widely used vegetation SIs is the normalized difference vegetation index (NDVI) [23]. Remote-sensing SIs can mitigate the effects of Sun angle, viewing angle, terrain, and atmospheric perturbations, and are therefore widely used to estimate crop parameters via remote sensing [24–28].

The last decades have seen the development of methods to estimate crop FVC based on remote-sensing images from unmanned aerial vehicle (UAV), aerial, or satellite platforms [5, 29–33]. These methods can be divided into five categories: (i) physical model methods, (ii) semi-empirical methods, (iii) empirical methods, (iv) crop growth methods, and (v) hybrid methods. Physical model methods are founded on physical principles; for example, the PROSAIL method, which is based on the optical properties of leaves and canopy bidirectional reflectance [15, 20, 34]; the four-scale bidirectional reflectance model, which is based on geometrical optics [35]; and the discrete anisotropic radiative transfer model, which is based on ray tracing [36–38]. However, many of the parameters required by these models may not be readily available, which limits the application of the models. Semi-empirical methods are often simplified versions of physical models and include the soil line method [39], the pixel dichotomy model (PDM) [40, 41], and the Baret model [29, 32]. The PDM hypothesizes that pixels contain mixed information from soils and crops [SI_total_ = (1 − FVC) × SI_soil_ + FVC × SI_vegetation_], which allows FVC to be calculated [FVC = (SI_total_ − SI_soil_)/(SI_vegetation_ − SI_soil_)] [42]. Empirical methods use remote-sensing SIs and regression techniques [e.g., linear and nonlinear (LAN) regression [43], partial least squares regression [44], random forest [45]] to establish an empirical model of FVC. Empirical methods usually provide good accuracy on a regional scale. Crop models were founded on crop-growth theory and provide FVC from sowing to harvest; these include the AquaCrop model [2] and the WOFOST model [3]. In addition to optical remote-sensing techniques, other remote-sensing techniques [e.g., synthetic aperture radar [30, 46]] have also been developed and applied to estimate FVC based on remote sensing. Hybrid methods involve the combined use of several of the methods mentioned above; for example, the model of Wang et al. [31, 47] uses crop modeling and remote-sensing-data assimilation. In recent years, the use of convolutional neural networks (CNNs) and high ground spatial resolution (GSD) images for estimating vegetation cover fractions has developed rapidly [48, 49]. The CNN-based studies were more focused on visual perception and image segmentation, instead of analyzing canopy spectral response to vegetation parameters (e.g., leaf inclination angle, leaf structure, pigments) [50, 51]. The training of CNN models involves a large number of samples. Furthermore, the application of CNNs is more suitable for high- and ultra-high-GSD images (e.g., digital images obtained from low altitude UAVs [48, 49], satellite-based high-GSD images [52]).

Two reasons explain why the PDM is widely used to estimate, based on remote-sensing images, cropland FVC from medium to high spatial resolution on the ground: (i) the results of the PDM have clear physical meaning and simple parameter input, and (ii) optical remote-sensing images with medium to high spatial resolution on the ground are available for free. The signal captured by each pixel in a remote-sensing image comes from a combination of soil background and vegetation of varying growth status (e.g., CCC, leaf water content, and LAI). In practice, the crop CCC is one of the key variables that determines the vegetation canopy spectral reflectance in the visible bands. For example, a high-CCC vegetation canopy corresponds to a large NDVI, whereas a low CCC vegetation canopy corresponds to a small NDVI. Thus, using the PDM on crop samples with low CCC may cause the FVC to be underestimated.

This study (i) analyzes how crop CCC affects SI-based estimates of FVC and (ii) estimates FVC for crops with various CCCs. To do this, we propose to use a fan-shaped method (FSM) that uses the visible and near-infrared angle index (VNAI) as SI for the CCC [53] and the NDVI as a vegetation SI to create a two-dimensional (2-D) scatter map in which the three vertices represent high-CCC vegetation, low-CCC vegetation, and soil. The FVC of each mixed pixel is determined based on its spatial locations in the 2-D scatter map, which weakens the dependence of the PDM on the CCC.

We use the proposed FSM and two traditional remote-sensing methods for estimating soybean FVC [i.e., (i) LAN regression an (ii) the PDM] based on spectra produced by applying the method to spectra obtained from (a) the PROSAIL model and (b) a spectrometer mounted on an UAV platform. The results show that the proposed FSM method can provide accurate estimates of FVC and may be applied in croplands with highly varying CCC.

## Methods

### Study site

**Fig. 1:**
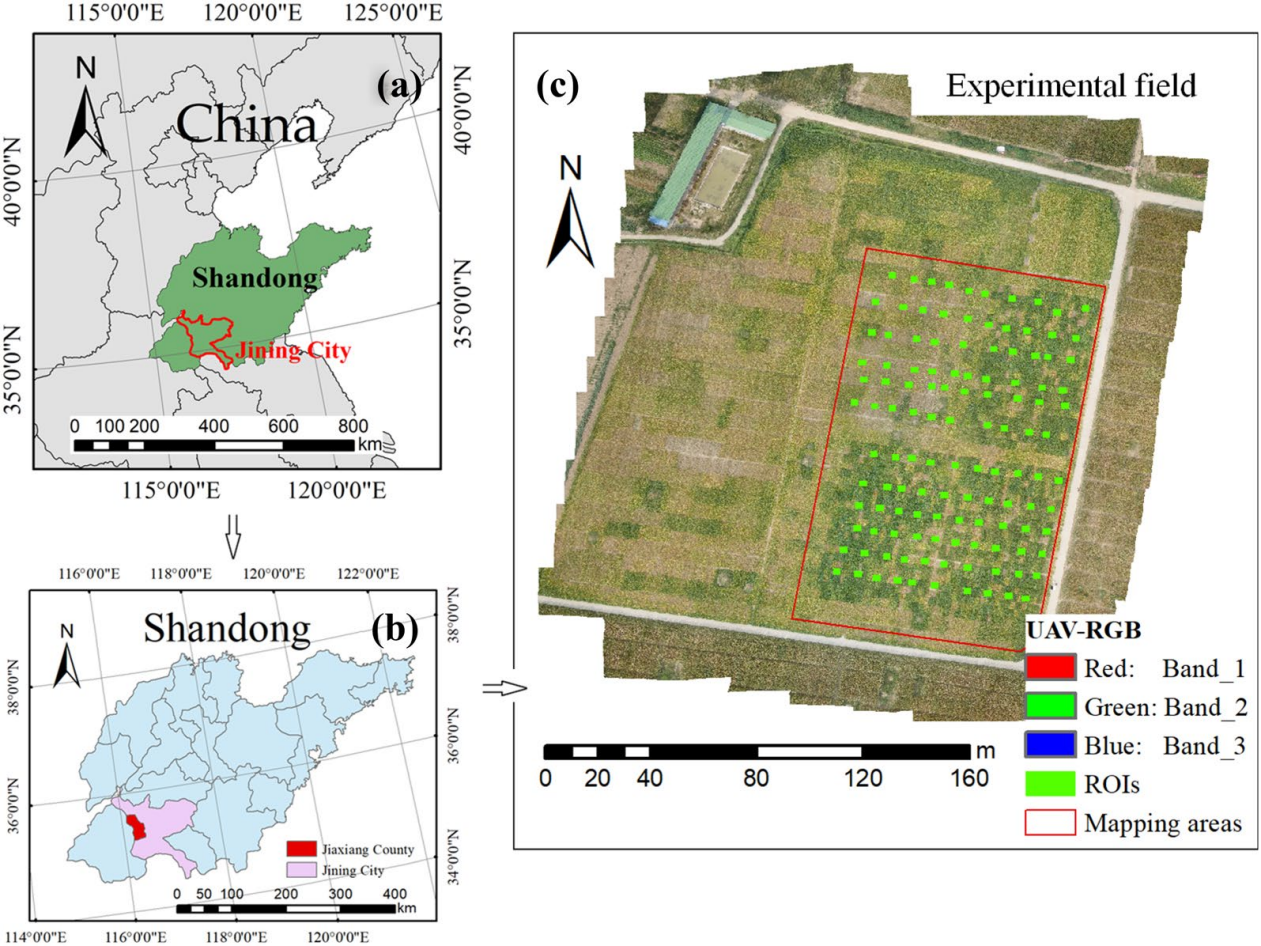
Study area and experimental field: **a** Jining City in Shandong province, China. **b** Location of Jiaxiang County in Jining City, Shandong province. **c** Mapping area and ROIs in experimental field (UAV-RGB image acquired September 17, 2015). Note: ROI is the region of interest, UAV stand for “unmanned aerial vehicle.”

The study site is situated in Jiaxiang County, Jining City, Shandong province, in China (see Fig. 1a, b). Jiaxiang County [Fig. 1b, E: 116°22′10″–116°22′20″, N: 35°25′50″–35°26′10″] has a warm temperate semi-humid continental monsoon climate, the average temperature is 13.9 °C, and the annual rainfall is 701.8 mm. Field experiments were conducted at a soybean field (see Fig. 1c). Soybeans were grown in a loam soil field with the row spacing of 15 cm, and the planting density of 190,000 plants ha^−1^. A total of 532 breeding lines were planted. Weed control was manually implemented at early growth stages.

### Measurement of field data

#### Measurements of field canopy chlorophyll content

The main purpose of field CCC measurements was to analyze the soybean CCC as a function of soybean growth. Soybean leaf chlorophyll in the first and second uppermost leaves was measured in the field by using a Dualex scientific portable sensor (Dualex 4; Force-A; Orsay, France) [54]. Five measurements of each soybean leaf were collected from the center of each soybean plot, and the average was retained as the soybean CCC (see Table 1). Forty-two soybean plots were selected for the field CCC measurements.

**Table 1:** Results of field measurements of soybean CCC (Dualex units)

Date (2015)	Stage and abbreviation	*n*	Min	Max	Mean	Standard deviation	Coefficient of variation	UAV
July 29	Flowering (S1)	42	23.19	33.84	26.82	2.42	9.01%	-
August 13	Early-podding (S2)	42	20.99	28.91	25.54	1.58	6.20%	-
August 31	Later-podding (S3)	42	29.27	47.83	37.38	3.01	8.07%	√
September 17	Grain-filling (S4)	42	6.52	38.28	25.92	8.18	31.58%	√
September 28	Harvest (S5)	24	8.81	36.05	21.33	7.62	35.77%	-
–	All stages	192	6.52	47.83	27.97	7.31	26.14%	-

Field soybean CCC measured by the Dualex 4 is marked as “Dualex units,” and *n* is the number of soybean plots. Some early-maturing plots were harvested during stage S5. Min, max and mean represent the minimum, maximum, and averaged value of soybean CCC

A total of 192 sets of soybean CCCs were collected from the soybean field from July 29 to September 28, 2015 (S1 to S5 in Table 1). Table 1 shows the results of the analysis of the CCC datasets. Overall, the average soybean CCC increases continuously from the flowering growth stage to the later-podding growth stage, and then decreases until harvest. The coefficients of variation calculated for the early stages S1–S3 are relatively small, 6.20%–9.01%. In contrast, the coefficients of variation calculated for the later stages S4–S5 are much larger, 31.58–35.77%.

#### Collection of UAV-based canopy RGB and hyperspectral images

The main purpose of UAV-based canopy digital images and spectral reflectance measurements is to analyze how soybean CCC affects FVC estimates based on remote-sensing images. The UAV flights were conducted during stages S3 and S4 (see Table 1). The hyperspectral and RGB images collected during stages S3 and S4 were used to analyze how CCC affects the soybean canopy spectral reflectance and SIs. The hyperspectral and RGB images collected during stage S4 were used to analyze how CCC affects FVC estimates.

In this work, UAV-based canopy RGB and hyperspectral images were collected from 11:00 a.m. to 2:00 p.m. from the soybean field before the field CCC dataset was collected. A DJI S1000 UAV was used as sensor platform (SZ DJI Technology Co., Ltd., Guangdong, China), on which was mounted a Sony DSC–QX100 digital camera (Sony, Tokyo, Japan) and a Cubert UHD-185 spectrometer (UHD 185, Cubert GmbH, Baden-Württemberg, Germany) to collect field crop-canopy RGB and hyperspectral images. We used a 40 cm × 40 cm whiteboard to calibrate the UHD-185 spectrometer before the UAV took off. The details of the UAV, UHD 185 snapshot hyperspectral sensor, and RGB camera are available in the literature [53, 55, 56].

The location of ground control points (GCPs) in the experimental field was determined by using a handheld Trimble GeoXT6000 global positioning system receiver. In this work, we used an Agisoft PhotoScan (Agisoft LLC, St. Petersburg, Russia) and soybean canopy digital images and hyperspectral images to generate the soybean canopy hyperspectral and RGB digital orthophoto maps (DOMs). After the hyperspectral and RGB images were stitched together, a RGB and a hyperspectral DOM for the experimental field were produced. The methods used to mosaic the hyperspectral and RGB images are available in the literature [56].

#### Extraction of canopy spectra and fractional vegetation cover

The UAV-based RGB and hyperspectral DOMs were pre-processed by using ENVI software (Exelis Visual Information Solutions, Boulder, CO, USA). A total of 120 regions of interests [ROIs, see Fig. 1c] were manually selected from the canopy image of the S4 stage. The following processing steps were involved:

(1) The UAV-based RGB DOMs were rectified by applying a field-measured GCPs in the ENVI software.

(2) Next, the UAV-based hyperspectral DOMs were rectified by using the UAV-based RGB DOM.

(3) The corresponding reflectance data were extracted from the hyperspectral DOMs by using the ENVI ROI tools.

From a UAV flying at an altitude of 50 m, the RGB camera can collect high-ground-resolution soybean canopy images (approximately 1.17 cm spatial resolution on the ground). Thus, almost all pixels contain pure leaf and background information. The following processing steps were done:

(1) Images of the selected 120 ROIs were classified by using the neural network classification tools in the ENVI software. Three labels were selected: soybean green leaf (soybean1), soybean yellow leaf (soybean2), and soil background;

(2) The number of pixels for soybean1 (*n*_soybean1_) and soybean2 (*n*_soybean2_) were counted for each ROI, and the FVC of each ROI was calculated by dividing the sum *n*_soybean1_ + *n*_soybean2_ by the total number *n*_total_ of each ROI [FVC = (*n*_soybean1_ + *n*_soybean2_)/*n*_total_].

This process produced a total of 120 sets of UAV-based canopy hyperspectral reflectance datasets and the corresponding FVC. Table 2 presents the statistical analysis of the FVC data from the 120 selected ROIs.

**Table 2:** Statistical analysis of FVC from 120 selected ROIs (*n* = 120, see Fig. 1)

Types	Number of plots	Minimum	Maximum	Average
Calibration	80	0.00	1.00	0.85
Validation	40	0.02	1.00	0.87
Total	120	0.00	1.00	0.86

### PROSAIL radiation-transfer model

The PROSAIL radiation-transfer model is widely used for analyzing how canopy reflectance is affected by leaf, canopies, and soil [14, 34, 57]. This work uses the PROSAIL model to analyze how CCC (Cab: 5:5:50 μg/cm^2^; see Table 1, minimum: 6.52, maximum: 47.83) and LAI (0.01, 0.5, 1, 1.5, 2, 3, 4, 6, 10) affect the canopy hyperspectral reflectance. The Cab and LAI parameters required special settings, whereas the other parameters were fixed (Table 3).

**Table 3:** Parameters of PROSPECT and SAIL

Models	Parameter	Symbol	Value or ranges	Units
PROSPECT	Leaf structure index	N	1.5	–
	Chlorophyll a + b content	Cab	5:5:50	μg/cm^2^
	Carotenoid content	Car	0	μg/cm^2^
	Brown pigments	Cbrown	0	–
	Equivalent water thickness	Cw	0.02	cm
	Dry matter content	Cm	0.01	g/cm^2^
SAIL	Leaf area index	LAI	0.01, 0.5, 1, 1.5, 2, 3, 4, 6, 10	m^2^/m^2^
	Hot spot effect	hspot	0.5	–
	Average leaf inclination angle	ALIA	45	
	Solar zenith angle	tts	20	
	Observer zenith angle	tto	0	
	Soil moisture factor	psoil	0.5	–
	Azimuth	psi	90	

– represents dimension-less variable

Table 3 lists the leaf and canopy parameters used as input for the PROSAIL model. In this work, the PROSAIL-based reference FVC (FVC_ref_) was calculated from the LAI by using the following relation between FVC and LAI [58, 59]:

1 $${\rm{FV}}{{\rm{C}}_{{\rm{ref}}}} = 1 - {e^{ - G \times \Omega \times \frac{{{\rm{LAI}}}}{{\cos \left( \theta \right)}}}},\;\;G = 0.5,\;\;\Omega = 1,\;\;\theta \; = 0,$$

where *G* is the leaf-projection factor for a spherical orientation of the foliage, Ω is the clumping index, *LAI* is the leaf area index, and *θ* is the viewing zenith angle. A simulation of the reflectance spectra of the vegetation canopy produced a total of 90 sets of spectra and FVCs (*n* = *n*_Cab_ × *n*_LAI_ = 10 × 9 = 90).

### Traditional remote-sensing method to estimate fractional vegetation cover

#### Linear and nonlinear regression

Previous studies have developed numerous vegetation SIs to estimate crop FVC. NDVI is a normalized transformation form of the NIR band and red band reflectance ratios. NDVI is defined as

2 $${\text{NDVI}} = \frac{{({R_{{\text{NIR}}}} - {R_{\text{R}}})}}{{({R_{{\text{NIR}}}} + {R_{\text{R}}})}},$$

where ${R_{{\text{NIR}}}}$ and ${R_{\text{R}}}$ are the vegetation canopy reflectances in the NIR and red bands, respectively. NDVI^2^ and the renormalized difference vegetation index (RDVI) [27] are two optimizations of NDVI. NDVI^2^ and RDVI are defined as

3 $${\text{NDV}}{{\text{I}}^2} = {\text{NDVI}} \times {\text{NDVI}},\;$$

4 $${\text{RDVI}} = \frac{{\left( {{R_{{\text{NIR}}}} - {R_{\text{R}}}} \right)}}{{{{\left( {{R_{{\text{NIR}}}} + {R_{\text{R}}}} \right)}^{0.5}}}}.$$

The soil-adjusted vegetation index (SAVI) [60] reduces the soil background effects:

5 $${\text{SAVI}} = \left( {1 + L} \right)\frac{{\left( {{R_{{\text{NIR}}}} - {R_{\text{R}}}} \right)}}{{\left( {{R_{{\text{NIR}}}} + {R_{\text{R}}} + L} \right)}},\;\;L = 0.5.\;$$

Many studies use LAN regression [43] to estimate vegetation FVC. These equations are

6 $${\text{FVC}} = a \times {\text{SI}} + b,$$

7 $${\text{FVC}} = a \times {\text{S}}{{\text{I}}^b},$$

where SI is a vegetation SI, and *a* and *b* are two empirical parameters to be obtained from the model calibration. We evaluate herein the results when using both the linear Eq. (6) and the exponential Eq. (7) to estimate vegetation FVC, but only the best FVC estimates (with the highest coefficient of determination, *R*^2^) are considered as LAN-based results.

#### Pixel dichotomy model

In the theory of linear spectral mixture analysis, the spectral element recorded in a mixed pixel combines the endmember spectra and their proportion. If a mixed pixel combines vegetation canopy and soil, the reflectance of band *i* can be expressed as

8 $${R_i} = {R_{i,{\text{veg}}}} \times {\text{FVC}} + {R_{i,{\text{\;soil}}}} \times \left( {1 - {\text{FVC}}} \right),$$

where *i* is the band number, *R*_*i*_ is the reflectance in band *i*, and *R*_*i,*veg_ and *R*_*i,*soil_ are the reflectances in band *i* from pure vegetation and pure soil, respectively. Similarly, the NDVI of a mixed pixel can be expressed as [40, 41]

9 $${\text{NDV}}{{\text{I}}_0} = {\text{NDV}}{{\text{I}}_{{\text{veg}}}} \times {\text{FVC}} + {\text{NDV}}{{\text{I}}_{{\text{soil}}}} \times \left( {1 - {\text{FVC}}} \right),$$

where ${\text{NDV}}{{\text{I}}_0}$ is the NDVI for mixed reflectance spectra, NDVI_veg_ and NDVI_soil_ are the NDVI of vegetation and soils. Then, FVC is calculated as

10 $${\text{FVC}} = \frac{{{\text{NDV}}{{\text{I}}_0} - {\text{NDV}}{{\text{I}}_{{\text{soil}}}}}}{{{\text{NDV}}{{\text{I}}_{{\text{veg}}}} - {\text{NDV}}{{\text{I}}_{{\text{soil}}}}}},$$

where FVC_veg_ and FVC_soil_ are the NDVI for vegetation and soils, respectively, and NDVI_0_ is the NDVI for mixed soil-vegetation reflectance spectrum.

### Proposed fan-shaped method

#### Visible and near-infrared angle index

**Fig. 2:**
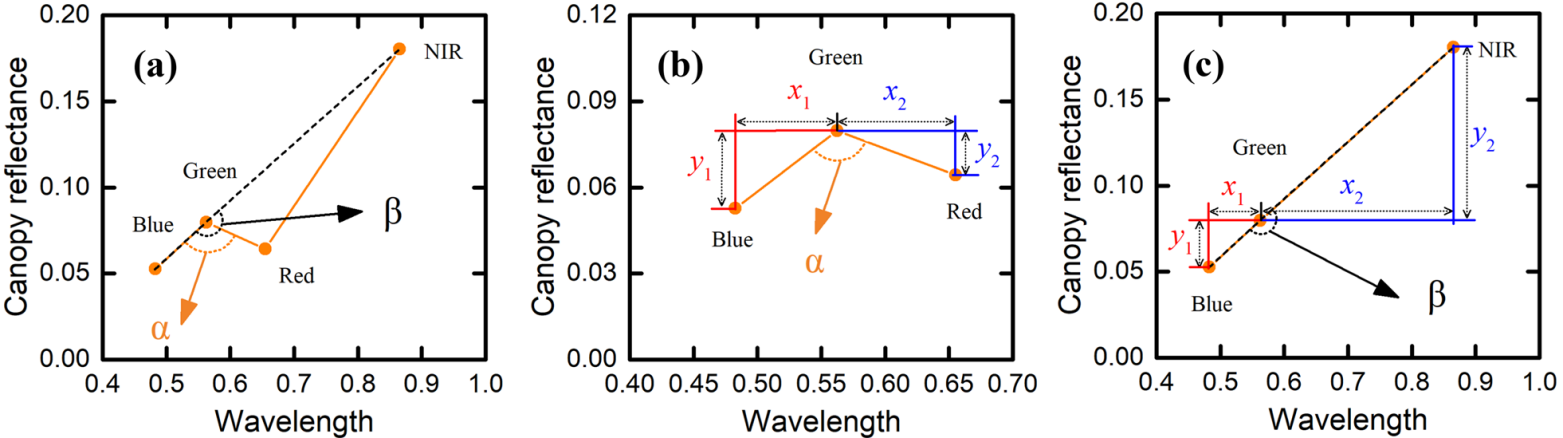
Calculation of angles α and β

We use a CCC SI to improve the FVC estimates based on the NDVI and PDM. The VNAI is a broadband optical CCC SI that uses the red, green, blue, and NIR bands (Fig. 2). As shown in Fig. 2(b), α is the angle enclosed by the rays G-B and G-R, and β is the angle enclosed by the rays G-B and G-NIR, and the VNAI can be explained as the sum of the two angles (VNAI = α + β) [53]. Yue (2020) shows that the VNAI can accurately estimate the CCC by relying on broadband remote-sensing reflectance as input.

Figure 2b and c show the method used to calculate the angles α and β. The result is

11 $${\text{angles}} = 180 - arctan\left( {\frac{y1}{{x1}}} \right) + arctan\left( {\frac{y2}{{x2}}} \right),$$

Mathematically, the angles can be calculated by using

12 $$\begin{gathered} \;\alpha = 180 - {\text{arctan}}\left( {\frac{{{R_{\text{G}}} - {R_{\text{B}}}}}{{{\text{wavelength}}\left( {{\text{G}} - {\text{B}}} \right)}}} \right) + {\text{arctan}}\left( {\frac{{{R_{\text{R}}} - {R_{\text{G}}}}}{{{\text{wavelength}}\left( {{\text{R}} - {\text{G}}} \right)}}} \right), \hfill \\ \;\beta = 180 - {\text{arctan}}\left( {\frac{{{R_{\text{G}}} - {R_{\text{B}}}}}{{{\text{wavelength}}\left( {{\text{G}} - {\text{B}}} \right)}}} \right) + {\text{arctan}}\left( {\frac{{{R_{{\text{NIR}}}} - {R_{\text{G}}}}}{{{\text{wavelength}}\left( {{\text{NIR}} - {\text{G}}} \right)}}} \right), \hfill \\ {\text{VNAI}} = \alpha + \beta , \hfill \\ \end{gathered}$$

where *R*_B_, *R*_G_, *R*_R_, and *R*_NIR_ are the spectral reflectance of the blue (492.4 nm), green (559.8 nm), red (664.6 nm), and NIR (832.8 nm) bands, respectively. The quantities (G–B) = (559.8–492.4)/2500 = 0.027, (R–G) = (664.6–559.8)/2500 = 0.0419, and (NIR–G) = (832.8–559.8)/2500 = 0.1092 represent the normalized distance (in wavelengths) covered by bands (i) G and B, (ii) R and G, and (iii) bands G and NIR, respectively. Note the ranges of ${\text{arctan}}\left( {\frac{{{R_{\text{G}}} - {R_{\text{B}}}}}{{{\text{wavelength}}\left( {{\text{G}} - {\text{B}}} \right)}}} \right)$, ${\text{arctan}}\left( {\frac{{{R_{\text{R}}} - {R_{\text{G}}}}}{{{\text{wavelength}}\left( {{\text{R}} - {\text{G}}} \right)}}} \right)$, ${\text{arctan}}\left( {\frac{{{R_{\text{G}}} - {R_{\text{B}}}}}{{{\text{wavelength}}\left( {{\text{G}} - {\text{B}}} \right)}}} \right)$, and ${\text{arctan}}\left( {\frac{{{R_{{\text{NIR}}}} - {R_{\text{G}}}}}{{{\text{wavelength}}\left( {{\text{NIR}} - {\text{G}}} \right)}}} \right)$ belong to (− 90°, 90°).

#### Visible and near-infrared angle index, spectral index, fan-shaped method

**Fig. 3:**
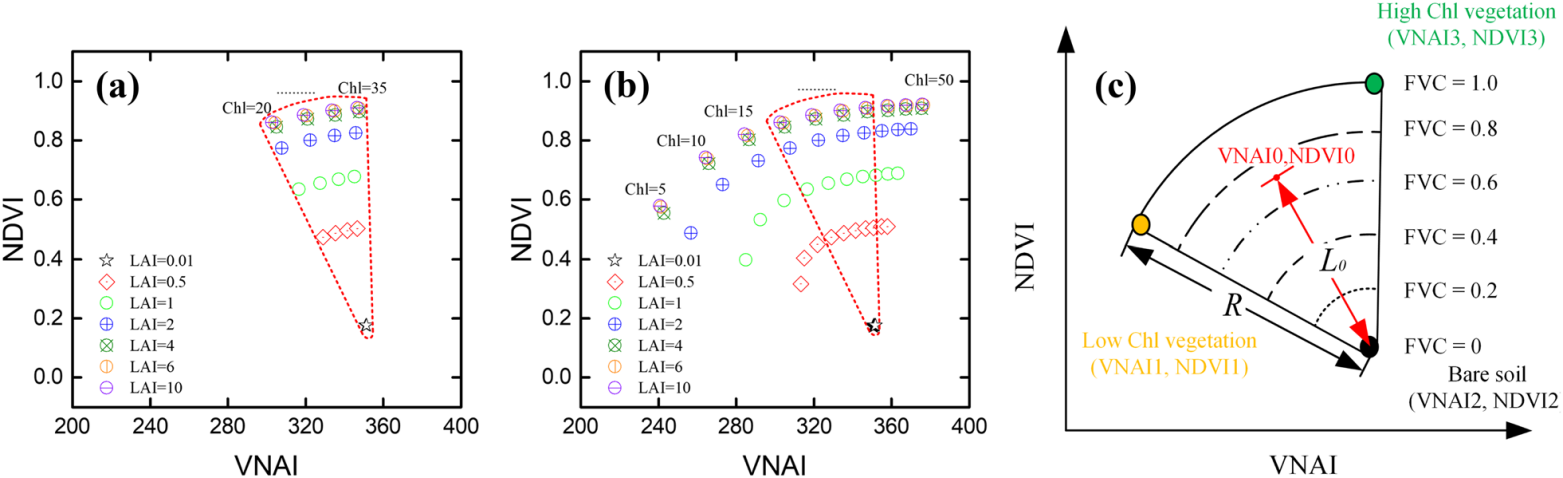
Theory for quantifying the fraction of high-CCC vegetation, low-CCC vegetation, and soil based on VNAI and NDVI: **a** PROSAIL-based NDVI as a function of VNAI (Cab = 20–35 μg/cm^2^). **b** PROSAIL-based NDVI as a function of VNAI (Cab = 5–50 μg/cm^2^). **c** Quantifying FVC using plot of NDVI vs. VNAI. Note: NDVI_1_, NDVI_2_, and NDVI_3_ are the NDVI values for low-CCC vegetation, bare soil, and high-CCC vegetation, respectively; VNAI_1_, VNAI_2_, and VNAI_3_ are the VNAI values for low-CCC vegetation, bare soil, and high-CCC vegetation, respectively; point (VNAI_0_, NDVI_0_) represents a mixed pixel on the VNAI–NDVI 2-D scatter map, NDVI is the normalized difference vegetation index, and VNAI is the visible and near-infrared angle index

We use the PROSAIL-based NDVI and VNAI to create a 2-D scatter map. As shown in Fig. 3(a, b), the optical-SIs for vegetation decrease with decreasing CCC. Figure 3(a) shows the 2-D scatter map for samples with medium-CCC (using 20–35 μg/cm^2^) and different FVC (i.e., different LAI). Figure 3(b) shows the 2-D scatter map for datasets (using 5–50 μg/cm^2^) containing low-, medium-, and high-CCC and different FVC (i.e., different LAI). As shown in Fig. 3(b)–(c), the proposed FSM uses the VNAI and NDVI to create a 2-D scatter map in which the three vertices represent high-CCC vegetation, low-CCC vegetation, and soil.

The FVC of each mixed pixel can be calculated as follows based on its location in the VNAI–NDVI fan-shaped 2-D scatter map (Fig. 3c):

13 $${\text{FVC}} = \frac{{L_0}}{r}$$

where *r* is the radius of the fan-shaped geometric figure and *L*_0_ is the distance from point (VNAI_0_, NDVI_0_) to the bare-soil point (VNAI_2_, NDVI_2_). Because the VNAI–NDVI 2-D scatter map is fan-shaped, the distance from the point for bare soil to low-CCC vegetation is the same as that to high-CCC vegetation, which is the radius of the fan-shaped geometric figure, thus

14 $$r = \sqrt {{{\left( {k \times {\text{VNA}}{{\text{I}}_3} - k \times {\text{VNA}}{{\text{I}}_2}} \right)}^2} + {{\left( {{\text{NDV}}{{\text{I}}_3} - {\text{NDV}}{{\text{I}}_2}} \right)}^2}} = \sqrt {{{(k \times {\text{VNA}}{{\text{I}}_2} - k \times {\text{VNA}}{{\text{I}}_1})}^2} + {{\left( {{\text{NDV}}{{\text{I}}_2} - {\text{NDV}}{{\text{I}}_1}} \right)}^2}} ,$$

where NDVI_1_, NDVI_2_, and NDVI_3_ are the NDVI values for low-CCC vegetation, bare soil, and high-CCC vegetation, respectively; VNAI_1_, VNAI_2_, and VNAI_3_ are the VNAI values for low-CCC vegetation, bare soil, and high-CCC vegetation, respectively; and the parameter *k* > 0 is the normalized distance from the VNAI to the NDVI. Thus, *k*^2^ is given by

15 $${k^2} = \frac{{{{\left( {{\text{NDV}}{{\text{I}}_2} - {\text{NDV}}{{\text{I}}_1}} \right)}^2} - {{\left( {{\text{NDV}}{{\text{I}}_3} - {\text{NDV}}{{\text{I}}_2}} \right)}^2}}}{{{{\left( {{\text{VNA}}{{\text{I}}_3} - {\text{VNA}}{{\text{I}}_2}} \right)}^2} - {{\left( {{\text{VNA}}{{\text{I}}_2} - {\text{VNA}}{{\text{I}}_1}} \right)}^2}}}$$

The FVC is then given by

16 $${\text{FVC}} = \frac{{L_0}}{r} = \frac{{\sqrt {{{\left( {k \times {\text{VNA}}{{\text{I}}_0} - k \times {\text{VNA}}{{\text{I}}_2}} \right)}^2} + {{\left( {{\text{NDV}}{{\text{I}}_0} - {\text{NDV}}{{\text{I}}_2}} \right)}^2}} }}{{\sqrt {{{(k \times {\text{VNA}}{{\text{I}}_3} - k \times {\text{VNA}}{{\text{I}}_2})}^2} + {{\left( {{\text{NDV}}{{\text{I}}_3} - {\text{NDV}}{{\text{I}}_2}} \right)}^2}} }},{\text{\;}}$$

17 $${\text{FVC}} = \frac{{\sqrt {{k^2}{{\left( {{\text{VNA}}{{\text{I}}_0} - {\text{VNA}}{{\text{I}}_2}} \right)}^2} + {{\left( {{\text{NDV}}{{\text{I}}_0} - {\text{NDV}}{{\text{I}}_2}} \right)}^2}} }}{{\sqrt {{k^2}{{({\text{VNA}}{{\text{I}}_3} - {\text{VNA}}{{\text{I}}_2})}^2} + {{\left( {{\text{NDV}}{{\text{I}}_3} - {\text{NDV}}{{\text{I}}_2}} \right)}^2}} }},$$

where NDVI_0_ and VNAI_0_ are the NDVI and VNAI value of a mixed pixel, respectively.

## Results and discussion

### Response of vegetation canopy reflectance spectra and spectral indexes to canopy chlorophyll content and leaf-area index

#### Response of canopy hyperspectral reflectance spectra and NDVI to canopy chlorophyll content and fractional vegetation cover

**Fig. 4:**
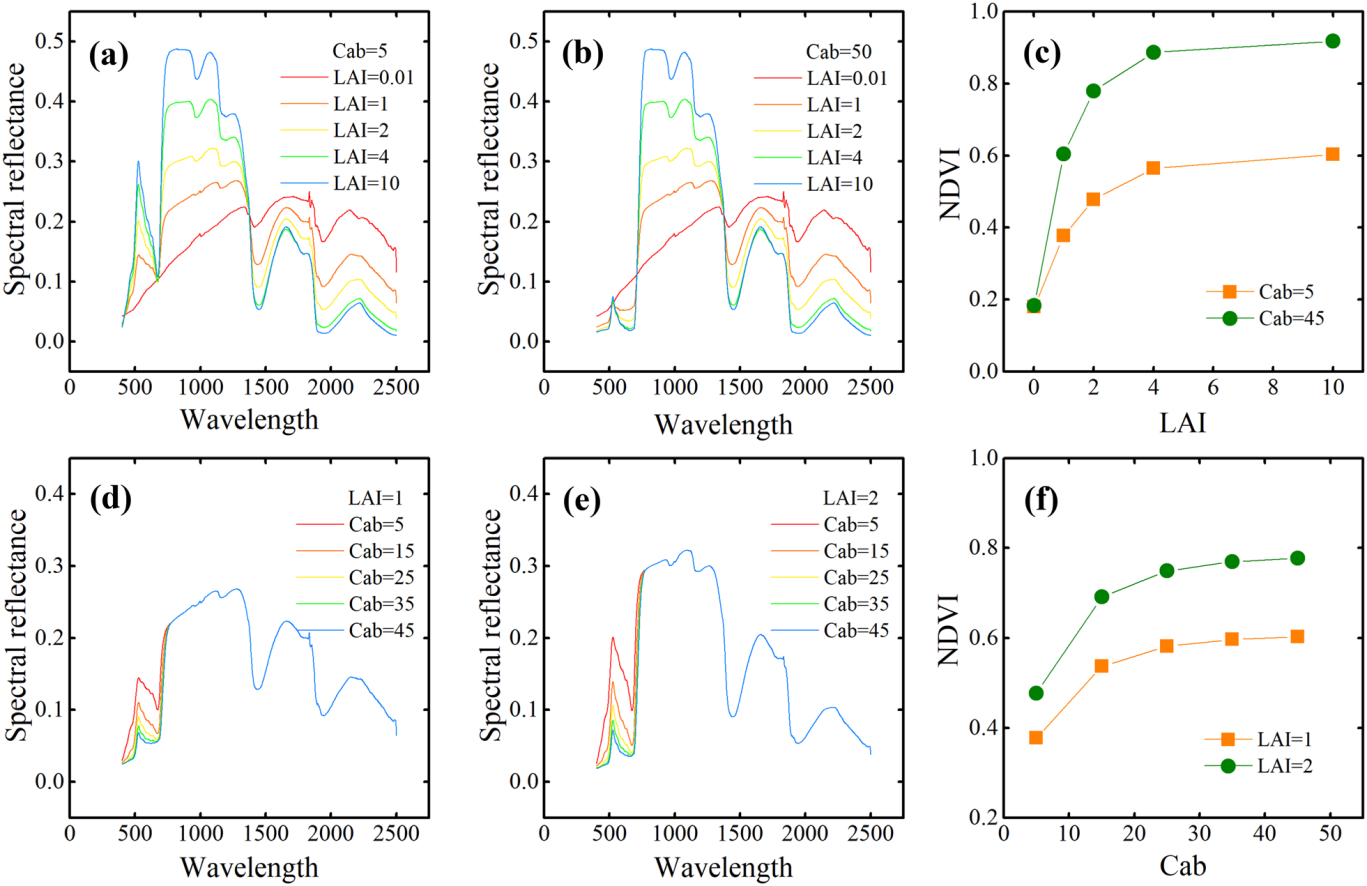
**a**, **b**, **d**, **e** Reflectance spectra of vegetation canopy and associated NDVI as a function of **c** LAI and **f** Cab. Note: Cab is the chlorophyll a and b content, LAI is the leaf-area index.

Figure 4 shows how vegetation canopy reflectance spectra and SIs depend on CCC (using Cab) and FVC (using LAI). As shown in Fig. 4(a–c), CCC affects the vegetation canopy reflectance spectra mainly in the visible and NIR bands (Fig. 4a, b). The canopy hyperspectral reflectance of high-CCC vegetation is less than that of low-CCC vegetation, and the NDVI of high-CCC vegetation exceeds that of low-CCC vegetation. The results shown in Fig. 4(d–f) also show that the NDVI of high-CCC vegetation exceeds that of low-CCC vegetation. Thus, the accuracy of multi-stage, SI-based FVC estimates is limited by variations in crop CCC (see coefficient of variation of CCC in Table 1).

**Fig. 5:**
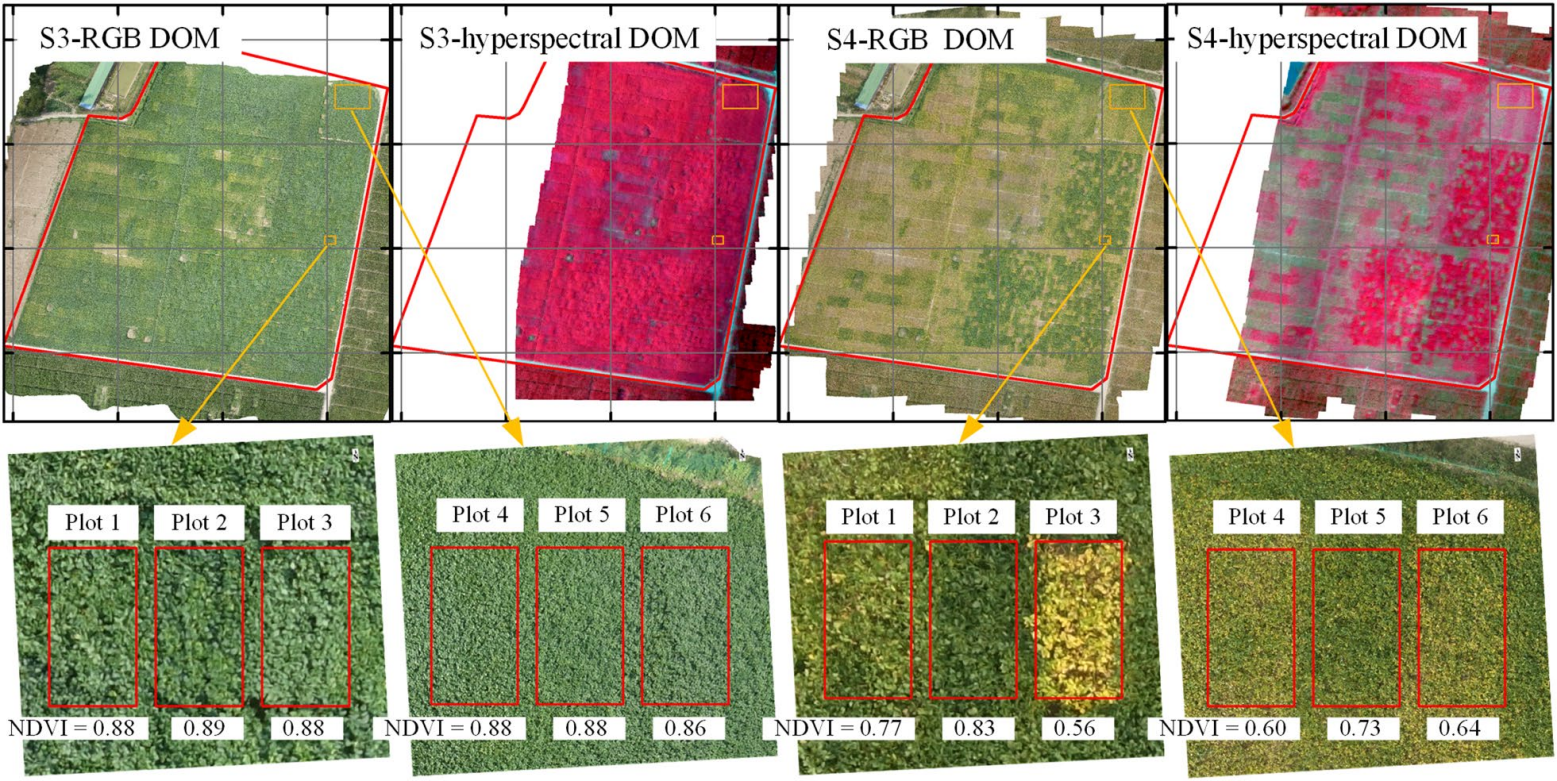
Dependence of hyperspectral images and RGB images (S3 and S4) on CCC. Note: UAV-based hyperspectral images are false-color images: R, G, B = 834, 662, and 558 nm, respectively. DOM stands for “digital orthophoto map.”

Figure 55 shows how UAV-based NDVI depends on CCC. FVC ≈ 1 for the six selected plots and two growth stages; the NDVI of six plots in stage S3 are also similar (from 0.86 to 0.89, see Fig. 5). However, the NDVI of the same six plant plots in the S4 stage differ significantly (from 0.56 to 0.83, see Fig. 5). Thus, the accuracy of multi-stage FVC estimation is limited by the variation of crop CCC (see Fig. 5).

Current methods for broadband remote-sensing FVC estimation are thus limited by vegetation CCC, principally because the optical SIs for pure crop canopies differ in the different growth stages. Many studies have concluded that the spectral reflectance in the visible bands and optical SIs for low-CCC vegetation canopies is lower than for high-CCC vegetation [16–18].

However, methods to reduce the effect of CCC on FVC estimation remain under-developed. In practice, the coefficient of variation of soybean CCC is huge in later growth stages (31.58%, Table 1), which, in turn, leads to lower optical SIs for low-CCC vegetation canopies than for high-CCC vegetation canopies. For example, the NDVI is high for high-CCC soybean (about 0.86–0.89, see Figs. 4 and 5), whereas the NDVI for low-CCC soybean is low (about 0.56, see Figs. 4 and 5). Thus, the LAN- and PDM-based methods may produce inaccurate estimates of FVC in the later growth stages, and FVC estimates based on data gathered over the long term depend essentially on the vegetation CCC.

#### How canopy chlorophyll content and fractional vegetation cover affect spectral indexes as a function of VNAI

**Fig. 6:**
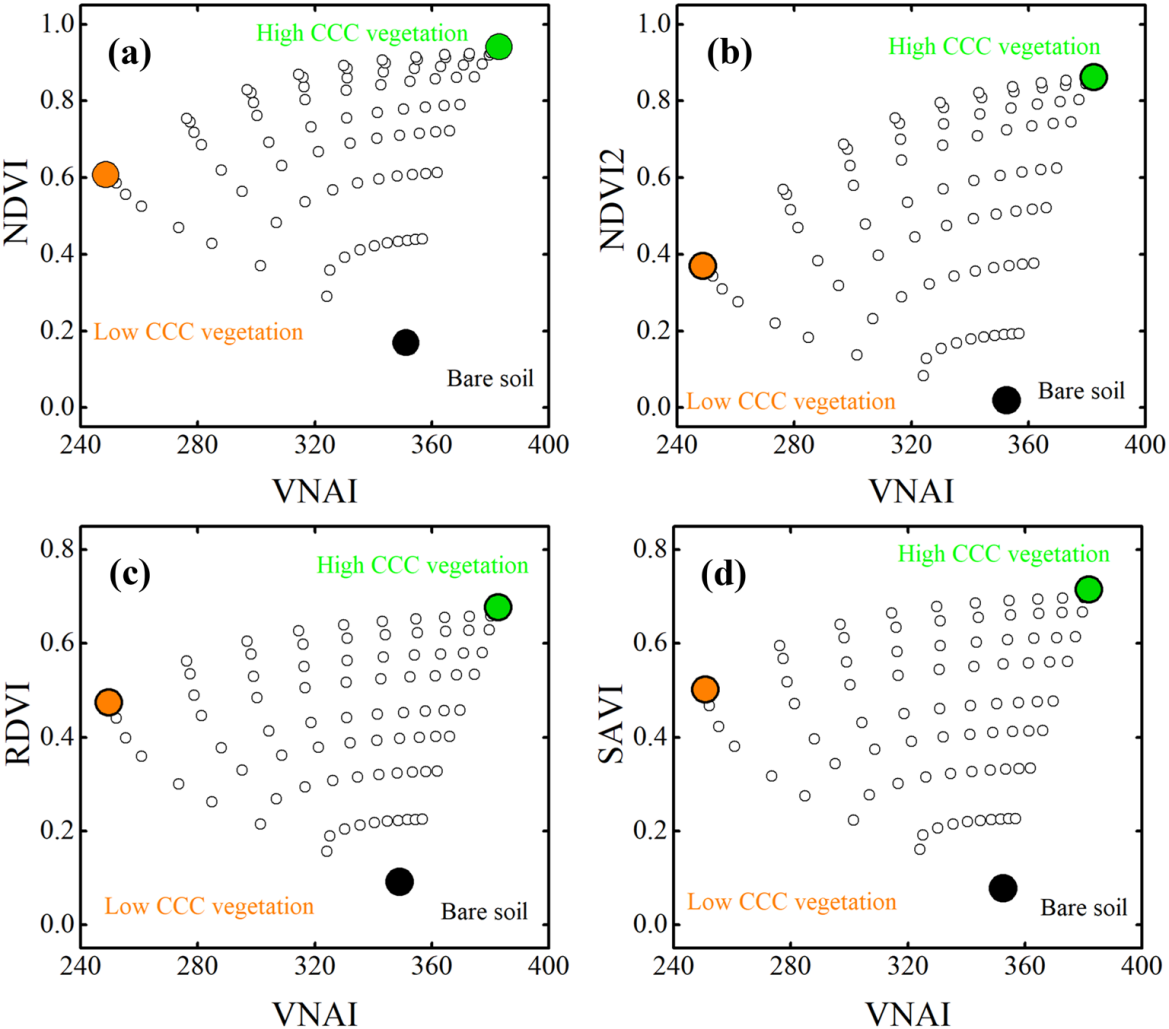
PROSAIL-based VNAI–SIs 2-D scatter maps. **a** VNAI–NDVI, **b** VNAI–NDVI^2^, **c** VNAI–RDVI, **d** VNAI–SAVI. *SI* spectral index, *RDVI* renormalized difference vegetation index, *SAVI*  soil-adjusted vegetation index

**Fig. 7:**
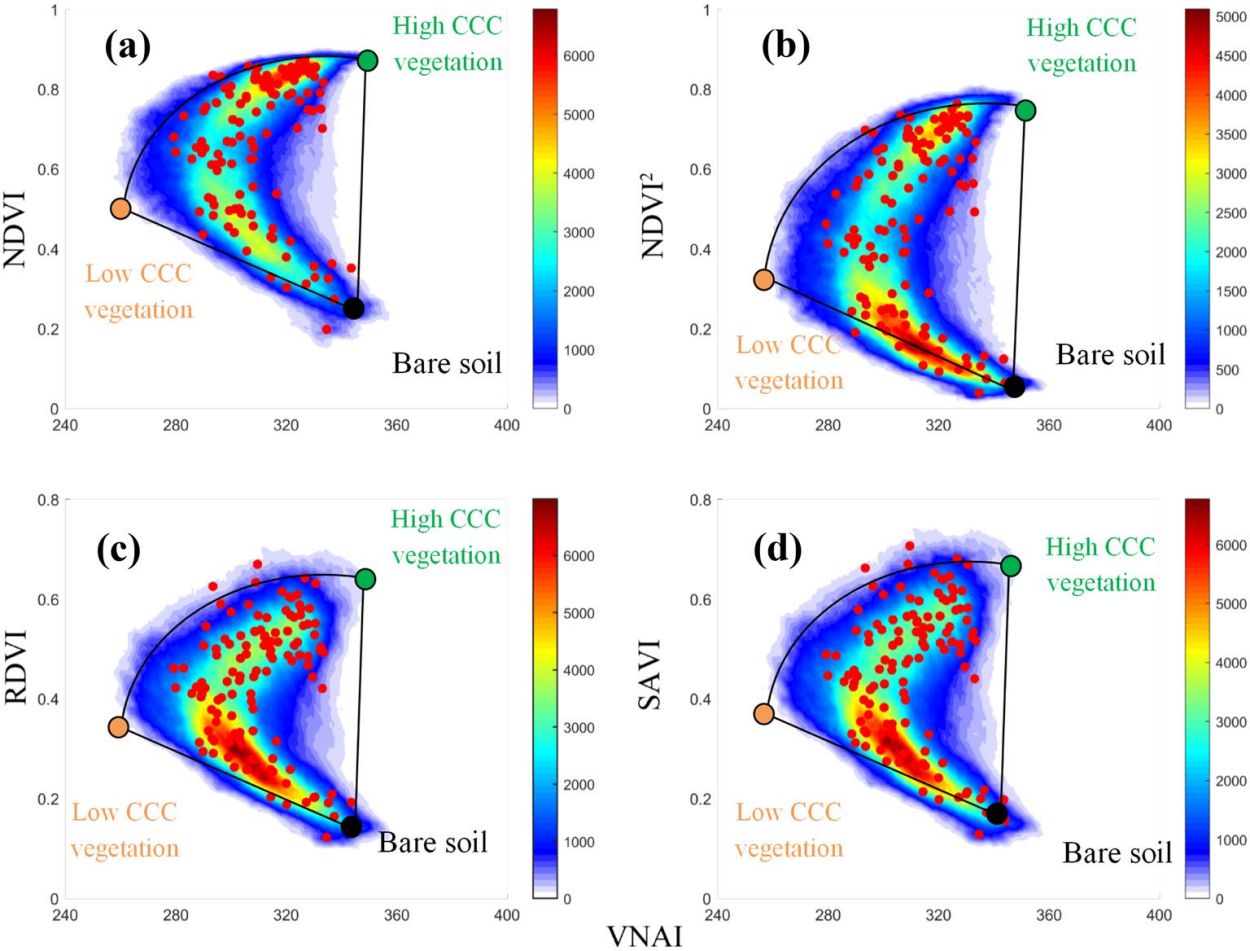
UAV-based VNAI–SI 2-D scatter maps for stage S4. **a** VNAI–NDVI, **b** VNAI–NDVI^2^, **c** VNAI–RDVI, **d** VNAI–SAVI. Note: Red dots represent the 120 ROIs from Fig. 1c

Figure 6 shows how CCC (using VNAI) and FVC (using NDVI) affects PROSAIL-based SIs as functions of VNAI. The VNAI–NDVI, VNAI–NDVI^2^, VNAI–RDVI, and VNAI–SAVI 2-D scatter maps are all similar: they form four fan-shaped 2-D scatter maps in which the three vertices represent high-CCC vegetation, low-CCC vegetation, and soil (see Fig. 3). The PROSAIL-based VNAI–SI 2-D scatter maps support our approach for quantifying the fraction of high-CCC vegetation, low-CCC vegetation, and soil based on a CCC SI and a vegetation SI. Figure 7 shows how CCC and LAI affect UAV-based SI vs. VNAI scatter maps. The UAV-based VNAI–NDVI, VNAI–NDVI^2^, VNAI–RDVI, and VNAI–SAVI 2-D scatter maps are similar for all PROSAIL-based simulations.

### Estimating and mapping fractional vegetation cover

#### Using LAN, PDM, and FSM to estimate fractional vegetation cover

**Fig. 8:**
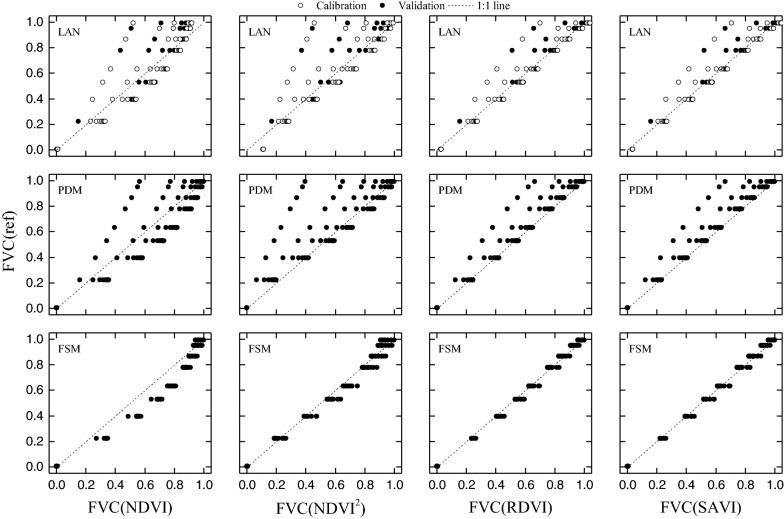
Reference FVC (FVC_ref_) and FVC estimated by using different SIs and methods (PROSAIL-based dataset). *LAN* linear and nonlinear regression, *PDM* pixel dichotomy model, *FSM* fan-shaped method. FVC_ref_ is the reference FVC calculated from Eq. (1)); FVC_NDVI_, FVC_NDVI2_, FVC_RDVI2_, and FVC_SAVI_ are FVC estimates based on **a** NDVI, NDVI^2^, RDVI, SAVI, and **b** LAN, PDM, and FSM

Figure 8 shows the reference FVC (FVC_ref_) and FVC estimated by using the methods LAN, PDM, and FSM and the SIs NDVI, NDVI^2^, RDVI, and SAVI. The accuracy of the FVC estimated by various models and SIs is listed in Table 3. The results suggest that the accuracy of FVC estimates made by LAN and PDM methods may be limited by variations in crop CCC. For example, given low CCCs, FVC is underestimated by LAN and PDM methods. In some extreme cases, using NDVI and LAN methods classify vegetation with 100% cover as having 50% cover (see Fig. 8). The most accurate FVC estimates are obtained by using the SAVI and the proposed FSM method (see Fig. 8 and Table 4, R^2^= 0.99, root-mean-square error (RMSE) = 0.03).

**Table 4:** Results of FVC estimates produced by various methods and based on various SIs (PROSAIL-based dataset)

Methods	Dataset	*n*	NDVI		NDVI^2^		RDVI		SAVI	
			*R*^2^	RMSE	*R*^2^	RMSE	*R*^2^	RMSE	*R*^2^	RMSE
LAN	Calibration	60	0.84	0.13	0.81	0.14	0.94	0.08	0.94	0.08
	Validation	30	0.80	0.15	0.77	0.15	0.92	0.09	0.93	0.08
PDM	Total	90	0.83	0.14	0.80	0.16	0.93	0.09	0.94	0.09
FSM	Total	90	0.95	0.11	0.98	0.05	0.99	0.03	0.99	0.03

The proposed FSM reduces the effect of the CCC by applying a PDM at each level of CCC (Fig. 3). For example (see Fig. 3b), the NDVI = 0.92 for high-CCC (Cab = 50) vegetation, the NDVI = 0.57 for low-CCC (Cab = 5) vegetation, whereas the NDVI = 0.17 for soil [FVC_(Cab = 5)_ = (NDVI _total_ – NDVI _soil_)/( NDVI _vegetation (Cab = 5)_ – NDVI _soil_), FVC_(Cab = 50)_ = (NDVI _total_ – NDVI _soil_)/( NDVI _vegetation (Cab = 50)_ – NDVI _soil_)]. The results of our PROSAIL-based estimates of the FVC (Fig. 8; Table 4) indicate that the proposed FSM method is robust and can be used for estimating FVC over multiple stages.

**Fig. 9:**
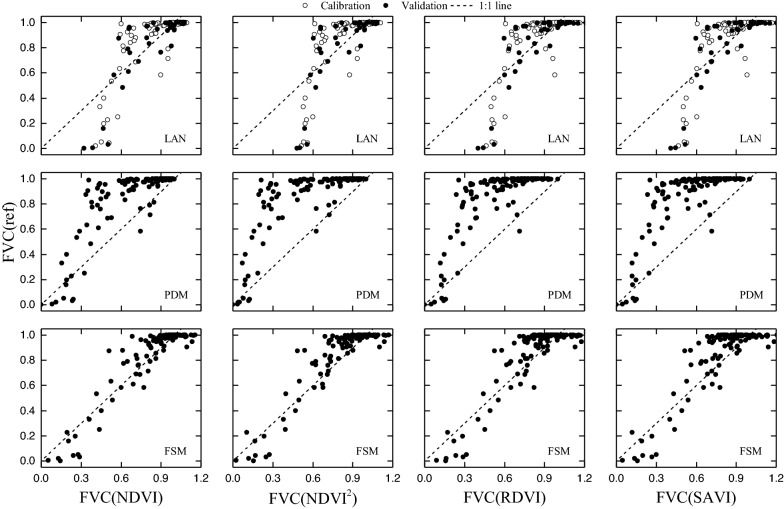
FVC measured and estimated by using different SIs and methods (UAV-based dataset). Note: FVC_ref_ is the reference FVC from UAV-image classification; FVC_NDVI_, FVC_NDVI2_, FVC_RDVI2_, and FVC_SAVI_ are the FVC estimates based on a NDVI, NDVI^2^, RDVI, SAVI, and **b** LAN, PDM, and FSM

Figure 9 shows the FVC measured and estimated by using different methods (LAN, PDM, and FSM) and SIs (NDVI, NDVI^2^, RDVI, and SAVI). The accuracy of the FVC estimates produced by different methods and SIs are listed in Table 4. The accuracy of FVC estimates based on the UAV-based dataset is similar to that of estimates calculated from the PROSAIL-based dataset (Fig. 8). FVC is underestimated by LAN and PDM when CCC is low. In some extreme cases, the PDM classifies vegetation with 100% cover as having 40% cover (see Fig. 7). The most accurate FVC estimates are obtained by using the FSM method (see Fig. 7; Table 5, total: *R*^2^ = 0.75–0.86, RMSE = 0.10–0.14). Thus, the results of UAV-based FVC estimates (see Fig. 7; Table 4) indicate that the proposed FSM method is a robust method that can be used to estimate FVC over multiple stages.

**Table 5:** Results of FVC estimates produced by various methods and based on various SIs (UAV-based dataset)

Methods	Dataset	*n*	NDVI		NDVI^2^		RDVI		SAVI	
			*R*^2^	RMSE	*R*^2^	RMSE	*R*^2^	RMSE	*R*^2^	RMSE
LAN	Calibration	80	0.61	0.16	0.51	0.17	0.54	0.17	0.54	0.17
	Validation	40	0.69	0.16	0.58	0.19	0.62	0.18	0.61	0.18
PDM	Total	120	0.64	0.23	0.54	0.32	0.57	0.34	0.57	0.34
FSM	Total	120	0.86	0.10	0.83	0.11	0.78	0.13	0.75	0.14

We evaluated the accuracy of FVC estimates based on a PROSAIL-based dataset, an image-based dataset, and the proposed FSM. Compared with LAN and PDM, the results indicate that FSM is a robust method that reduces the influence of crop CCC and thereby provides the most accurate estimates of FVC. As shown in Figs. 8 and 9, the FVC of samples with low CCC are underestimated by the PDM; and the LAN method underestimates the FVC of low-CCC samples and overestimates the FVC of high-CCC samples. By using a CCC SI, the proposed FSM mitigates the effect CCC and thereby improves FVC estimates. Considering that the variation of CCC is one of the most influential factors in the PDM (Figs. 4, 5), the proposed FSM offers the advantage of accurately estimating FVC over multiple stages.

#### Mapping fractional vegetation cover using image-based dataset

**Fig. 10:**
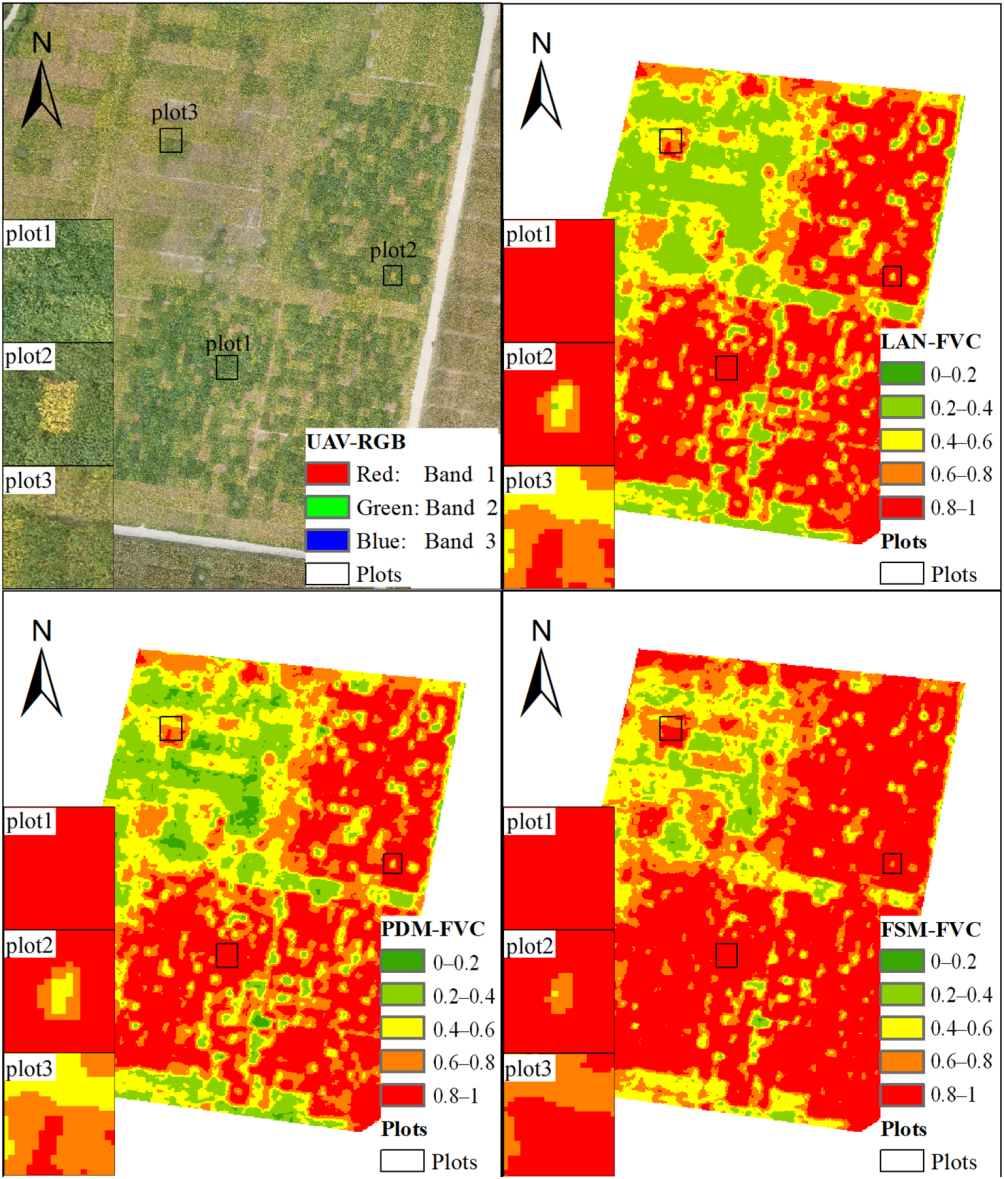
FVC mapping. Note: Plot 1: high-CCC and high FVC; Plots 2 and 3: low-CCC and high FVC

The FVC maps were calculated by using (i) the NDVI based on UAV-hyperspectral images and (ii) LAN, PDM, and FSM. Figure 10a–d show the UAV RGB DOM, the LAN-FVC map, the PDM-FVC map, and the FSM-FVC map. As shown in Fig. 10, three plots were selected for performance evaluation. Similar to the PROSAIL-based results shown in Fig. 4, the LAN, PDM, and FSM estimates of FVC for plot 1 (high-CCC plot: green leaves) are similar; however, the FVC of plots 2 and 3 (low-CCC plots: yellow leaves) are underestimated by LAN and PDM. Thus, the results shown in Fig. 10 suggest that the FVC map calculated by the proposed FSM is more accurate.

In this work, soybean hyperspectral images acquired at later growth stage were used for validation. These images cover low-, medium-, and high-CCC soybean plots (Fig. 10). The CCC and LAI are the dominating factors affecting the visible bands of vegetation canopy spectra. Consequently, the estimation of FVC at early growth stages [CCC: about 20–35 μg/cm^2^, see Fig. 3a, red fan-shape] is similar to that for median-CCC samples at later growth stages when using the PROSAIL-based dataset [see Fig. 3b, red fan-shape]. In this study, no field FVC and real canopy spectra for early stages were tested. Further quantitative validation from field sites is necessary.

The most significant advantage of the FSM is that it can be used to estimate and map crop FVC from various CCC conditions. As shown in Table 1, the coefficient of variation of soybean CCC is small during the early growth stages but increases significantly in the later growth stages. Thus, to estimate FVC for mapping in the later growth stages and over multiple growth stages, one should consider the variation of crop CCC (Figs. 5, 6, 7). We use the proposed FSM to calculate FVC at different CCC levels, which may help to provide FVC estimates that are more robust than those provided by PDM and LAN. However, as with any method, the FSM has shortcomings, the most obvious of which is that it requires an additional parameter [*k*^2^, see Eqs. (13)–(16)] to normalize the distance from the VNAI to the NDVI, which may limit its application. As shown in Eq. 15, the parameter *k*^2^ depends on the NDVI and VNAI of soil, high CCC vegetation, and low CCC vegetation. But in practice, the NDVI and VNAI values for different soybean cultivars differ due to leaf and canopy parameters (see Table 3, leaf structure, carotenoid content, etc.). Thus, the parameter *k*^2^ needs to be calibrated for practical application of FSM (see Eq. 15).

The strategy of combining a CCC SI into the FSM could also be applied to satellite multispectral remote-sensing imagery, but the feasibility of FVC estimates based on long-term data acquired from satellite multispectral remote-sensing images remains to be validated. In this work, the proposed FSM was validated by using only PROSAIL-based simulations and a UAV-based soybean canopy spectral image from a single site. Thus, further validation is required from additional crops and study sites.

## Conclusions

To estimate FVC, we propose herein a FSM that mitigates the effect of CCC by conducting a PDM at each CCC level. The FSM is a robust method that can be used to estimate FVC based on multiple growth stages where crop CCC varies greatly. The results lead to the following two conclusions:

1. Estimates and maps of FVC based on the later growth stages and on multiple growth stages should consider the variation of crop CCC. Field soybean CCC measurements (Table 1) indicate that (a) the soybean CCC increases continuously from the flowering growth stage to the later-podding growth stage, and then decreases with increasing crop growth stages, (b) the coefficient of variation of soybean CCC is very large in later growth stages (31.58–35.77%) and over all growth stages (26.14%). Thus, PDM underestimates the FVC of samples with low CCC, and LAN underestimates the FVC of low-CCC samples and overestimates the FVC of high-CCC samples.
2. The proposed FSM method can provide accurate FVC estimates based on data from multiple growth stages and can be applied to croplands with significant variation in crop CCC. The proposed FSM mitigates the influence of CCC by applying a PDM at each CCC level, making it a robust method for multi-stage estimates of FVC in situations involving strong variations in crop CCC. The improved FVC estimates are validated by both PROSAIL- and images-based datasets.

## Data Availability

The datasets used and/or analyzed during the current study are available from the corresponding author on request.

## Abbreviations

2-D: Two-dimensional
CCC: Canopy chlorophyll content
CNN: Convolutional neural network
DOM: Digital orthophoto map
FSM: Fan-shaped method
FVC: Fractional vegetation cover
FVC_ref_: Reference FVC
GCP: Ground control point
GSD: Ground spatial resolution
LAI: Leaf-area index
LAN: Linear and nonlinear regression
NDVI: Normalized difference vegetation index
NIR: Near-infrared
PDM: Pixel dichotomy model
RDVI: Renormalized difference vegetation index
RMSE: Root-mean-square error
SAVI: Soil-adjusted vegetation index
SI: Spectral index
UAV: Unmanned aerial vehicle
VNAI: Visible and near-infrared angle index
